# Comparison of transabdominal wall specimen retrieval and natural orifice specimen extraction robotic surgery in the outcome of colorectal cancer treatment

**DOI:** 10.3389/fsurg.2023.1092128

**Published:** 2023-02-16

**Authors:** Ju Houqiong, Wan Ziwen, Zhong Chonghan, He Penghui, Yu Hongxin, Lu Weijie, Liu Dongning, Li Taiyuan

**Affiliations:** ^1^ Department of General Surgery, The First Affiliated Hospital of Nanchang University, Nanchang, China; ^2^ Laboratory of Digestive Surgery, Nanchang University, Nanchang, China; ^3^ The First Clinical Medical College of Nanchang University, Nanchang, China

**Keywords:** robotic surgery, natural orifice specimen extraction, quality of life, colorectal cancer, survival

## Abstract

**Background:**

Natural orifice specimen extraction surgery (NOSES), as a new star of minimally invasive techniques, has been increasingly favored and promoted in the field of surgery around the world. Most previous studies were comparative studies of laparoscopic NOSES and conventional laparoscopic surgery. However, there is little research on comparing robotic colorectal cancer NOSES with conventional robotic-assisted colorectal cancer resection surgery.

**Participant and methods:**

This study is a retrospective study of propensity score matching (PSM). This study included Ninety-one propensity score-matched pairs of the participant who had undergone robotic colorectal cancer resection surgery at our center between January 2017 and December 2020. The covariates used in the propensity score included gender, age, BMI, ASA score, maximum tumor diameter, the tumor's height from the anal verge, histological differentiation, AJCC stage, T stage, N stage, and history of previous abdominal surgery. The outcome measurement criteria included postoperative complications, inflammatory response, pelvic floor function, anal function, cosmetic outcome, quality of life, disease-free survival (DFS), and overall survival (OS).

**Results:**

The robotic NOSES group had faster recovery time from gastrointestinal function (*P* = 0.014), shorter abdominal incision length (*P* < 0.001), less pain (*P* < 0.001), less additional analgesia required (*P* < 0.001), and lower postoperative indicators of white blood cell count (*P* < 0.001) and C-reactive protein content compared to the robotic-assisted resection surgery (RARS) group (*P* = 0.035). Additionally, the robotic NOSES group had significantly better body imagery (*P* < 0.001), cosmetic scores (*P* < 0.001), somatic function (*P* = 0.003), role function (*P* = 0.039), emotional function (*P* = 0.001), social function (*P* = 0.004), and overall function (*P* < 0.001) than the RARS group. The two groups demonstrated no significant difference between DFS and OS.

**Conclusion:**

Robotic colorectal cancer NOSES is a safe and feasible minimally invasive procedure and offers shorter abdominal incisions, less pain, less surgical stress response, and better postoperative quality of life. Therefore, this technique can be further promoted for colorectal cancer patients eligible for NOSES.

## Introduction

Colorectal cancer (CRC) is the third-leading malignant tumor and the second-highest contributor to cancer deaths globally (1). The current treatment protocol for CRC is still a multidisciplinary and comprehensive diagnosis and treatment model based on radical surgery (2). Since 1991, the world's first laparoscopic rectal cancer surgery, which is representative of minimally invasive surgery because of its minor trauma, rapid recovery, and long-term treatment of tumors, is not different from open surgery, with rapid promotion and application (3–5).

The unique advantages of the surgical robot make colorectal surgery operations more precise and intelligent, providing more options for minimizing invasion of the colorectum. A multicenter, large-sample, randomized controlled study in China (6) and several meta-analyses (7–9) revealed that robotic surgery for colorectal cancer has better lumpectomy quality and demonstrates an oncology outcome similar to conventional laparoscopic surgery for the long term.

However, conventional robotic or laparoscopic surgery requires an additional incision to complete specimen removal. The abdominal wall incision is the most direct and compelling evidence of the minimally invasive outcome of the procedure. Additionally, adjuvant abdominal wall-assisted incisions are associated with an increased potential for postoperative incision-related complications, composed of wound infections, incisional hernias, and even incisional implant metastases (10, 11). The minimally invasive natural orifice specimen extraction surgery (NOSES) has tackled this complication. Moreover, NOSES can provide good near-term outcomes while satisfying the need for radical tumor treatment (12–14). The combination of surgical robots and NOSES can bring incredible benefits to colorectal cancer patients.

The proximity between the sigmoid column and rectum to the anal location provides a favorable predisposition for transanal specimen retrieval without significantly increasing the difficulty of the surgical operation. Moreover, few studies compared robotic colorectal cancer NOSES with conventional robotic-assisted colorectal cancer resection surgery (RARS). Hence, participants who underwent robotic sigmoid or upper segment of rectum cancer surgery at our center were selected for this study. The safety and feasibility of robotic colorectal cancer NOSES surgery were further confirmed by comparing and analyzing the short- and long-term outcomes of robotic colorectal cancer NOSES with those of conventional robotic colorectal cancer surgery. This study aimed to provide more reliable and accurate proof of clinical evidence for robotic colorectal cancer NOSES.

## Material and methods

### Study population and comparative group

The study was a retrospective single-center team-based study conducted at First Affiliated Hospital of Nanchang University. All procedures were per the moral criteria of the Center and the Declaration of Helsinki. The study included all recipients of respectable sigmoid and upper segment rectal cancers without distant metastases diagnosed by imaging and histology in the Department of Gastrointestinal Surgery at our center and undergoing radical robotic resection in the period beginning January 2017 through December 2020.

Inclusion criteria: (1) postoperative pathologically confirmed colorectal adenocarcinoma; (2) tumor located in the sigmoid colon, recto-b junction, and upper rectum (lower edge of tumor located above the peritoneal reflex) according to imaging, colonoscopy, intraoperative findings, and postoperative pathology; (3) patient's body mass index (BMI) <35 kg/m^2^; (4) no distant metastasis according to preoperative examination and intraoperative findings.

Exclusion criteria: (1) concurrent combination of other malignancies; (2) emergency surgery for bleeding, obstruction, or perforation; (3) patients receiving preoperative radiotherapy; (4) a lack of follow-up data or incomplete data; (5) stoma prophylaxis patients or patients with stoma caused by other reasons.

The inclusion and exclusion criteria for both groups were referred to in the criterion mentioned above. Ultimately, 282 patients were included. Among them, 138 patients undergoing robotic natural orifice specimen extraction surgery were in the NOSES group, and 144 who underwent conventional robotic-assisted resection surgery were in the RARS group.

### Surgical technique

In the NOSES and RARS groups, successful anesthesia was followed by intraoperative confirmation of no distant metastases. Colon cancer surgery and rectal cancer surgery were performed according to complete mesocolic excision (CME) and total mesorectal excision (TME), respectively. Surgery was performed by the same surgeon who had received standardized training in robotic surgery and completed over 200 colorectal cancer procedures.

In the RARS group, the inferior mesenteric vessels were carefully dissected, the lymphatic adipose tissue was cleared, and the rectum or colonic mesentery was freed, with at least 2 cm from the below edge of the tumor, the mesentery naked, and the closed rectum cut. The adjacent organs and pelvic autonomic nerves must be protected during the operation. A minor incision was taken to remove the specimen from the lower abdomen, and a reconstruction of the digestive tract was excised and completed. Both sigmoid and rectal cancer resections were performed with a circular stapler for end-to-end anastomosis.

**Figure 1:**
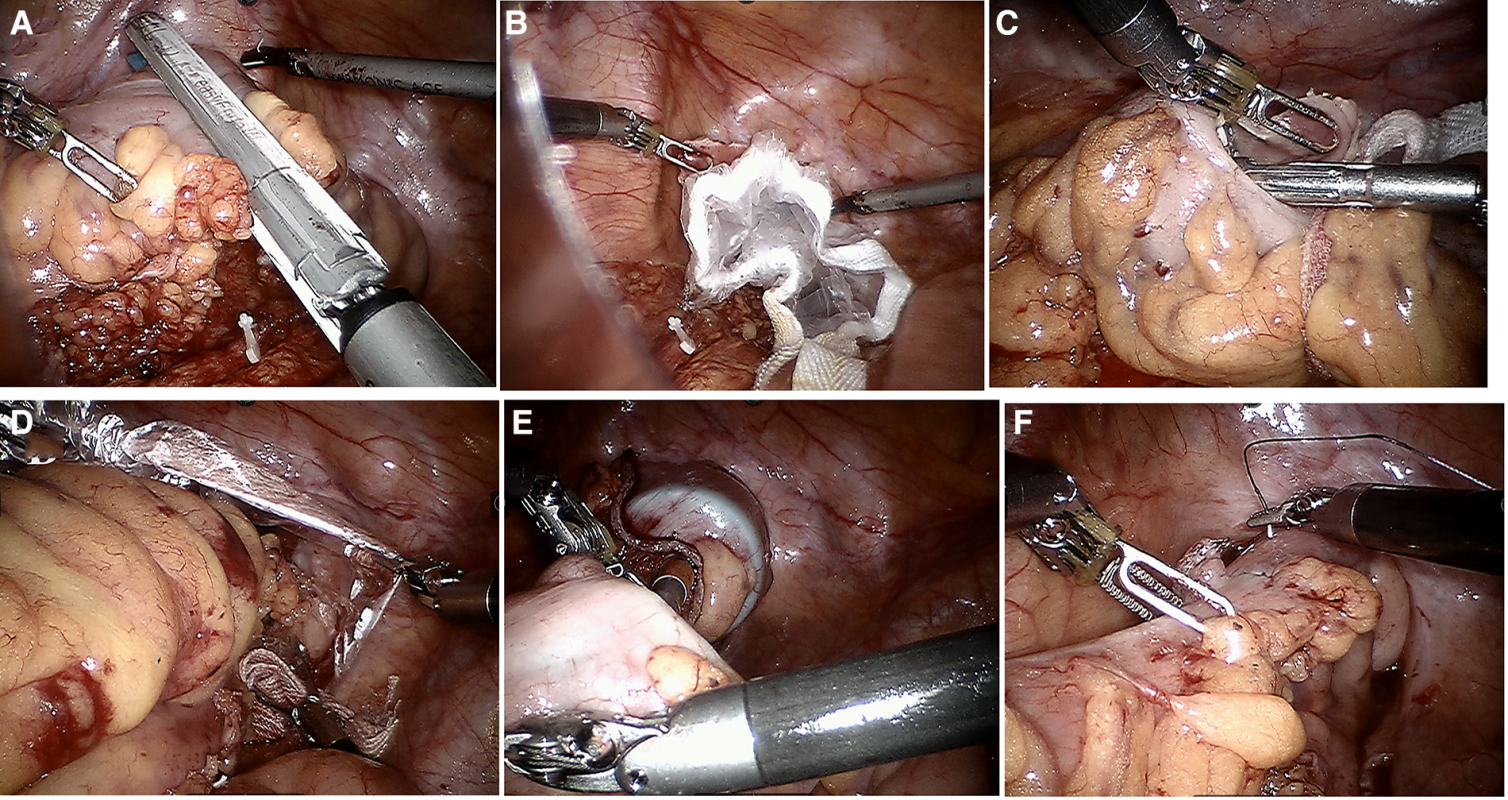
Key surgical steps of natural orifice specimen extraction surgery (**A–F**). (**A**) Excisional occluder to dissociate tumor specimens; (**B**) sterile protective sleeve is placed into the rectum to establish sterile access; (**C**) staple holder placed into the proximal rectum; (**D**) transanal removal of the specimen; (**E**) end-to-end sigmoid-rectal anastomosis; (**F**) intraluminal reinforced anastomosis.

In the NOSES group, there was no difference from the conventional robot in freeing the intra-abdominal tract, lymph node dissection, and tumor resection. However, differences were observed in the route of specimen removal and gastrointestinal tract reconstruction. Transrectal specimen extraction for sigmoid and upper rectal cancer was NOSES IV. The procedure consisted of sufficient mild dilation and transanal flushing of the rectum cavity by iodine saline. The rectum was dissected by a linear stapler at 4 cm away from the lower margin of the tumor, while a sterile protected package was placed through the assistant hole and dragged out of the anus through the rectum. The staple holder was held with a toothed clamp and delivered to the abdominal cavity through the sterile-protected package. At 3 cm below the sigmoid nakedness, the intestinal canal was incised with an ultrasonic knife. The staple holder was disposed into the intestinal cavity of the sigmoid colon through the incision and pushed into the proximal colon until the connecting rod crossed the sigmoid nakedness. After gently straightening the wire, the sigmoid colon was disconnected with a linear stapler at the sigmoid colon tube nudity. The specimen was dragged out of the body *via* the anus with toothed forceps placed in the protective sleeve. The rectal stump was closed with a linear stapler. Dissection was performed at the sigmoid colon to find the broken end of the silk thread. Then, the staple holder was dragged out distally by tugging on the silk thread, and the outline of the staple holder rod was seen. Afterward, the staple holder rod was pulled out through the silk thread, and the pelvic cavity was rinsed with iodine saline. Active bleeding was checked. Subsequently, gastrointestinal reconstruction was performed to end-to-end anastomose the descending colon and rectum with a circular stapler. The end-to-end anastomosis of the descending colon and rectum was completed by placing a loop anastomosis clutch through the anus after the absence of sigmoid mesenteric torsion was determined. The gas injection test checks whether there is leakage in the anastomosis, and the dangerous triangle of the anastomosis is stitched with the assistance of the robot. The critical operation for transrectal specimen retrieval is illuminated in Figure 1.

### Data collection and follow-up

Data collection. Each case was requested to report demographic information, preoperative diagnosis, operational state, postoperative pathology, postoperative complications, recovery information, and long-term follow-up information. Overall survival (OS) was defined as the time from surgery to death from any cause. Disease-free survival (DFS) was defined as the time from surgery to disease recurrence or death from any cause. (1) Patients were asked about their attitudes toward their physical appearance and how satisfied they were with the scar's appearance 1 month after surgery. The Body Imagery Questionnaire (BIQ) was used in cholecystectomy (15) and nephrectomy patients (16). (2) A PFDI-20 score was employed to assess patients’ symptoms 6 months after surgery, including urinary tract symptoms, gastrointestinal symptoms, and pelvic organ prolapse (17). (3) Patient Scar Assessment Questionnaire and Scoring System (PSAQ) was adopted to assess the cosmetic outcome of patients at 3 months postoperatively, as well as the outcome of any linear scar surgical treatment (18). (4) EORTC QLQ—C30 scale was utilized to assesses patients’ quality of life at 3 months postoperatively (19). (5) After 6 months after surgery, patients were assessed for incontinence using the Wexner incontinence score (20).

Follow-up. Routine follow-up was scheduled 1 month after surgery by National Comprehensive Cancer Network guidelines, every 3 months for 2 years, and every 6 months for 5 years until patient death or study termination. The information was available by e-mail or telephone if the participant did not return for observation. All patients were followed until death or September 31, 2022.

### Statistical analysis

Since the propensity score matching method (PSM) can minimize selectivity bias, it was used to counterbalance the baseline information among the two groups. PSM was matched at a ratio of 1:1 for propensity scores against the above baseline information. A logistic regression model was applied to assign these variables to the baseline information among 282 patients with a caliper value of 0.02. The measures conformed to a normal distribution and were presented as mean ± SD. The measures that were not normally distributed were performed using independent samples *t*-test or Mann-Whitney *U* test, indicated as median and quartiles, respectively. Count data were presented as frequencies and percentages using the *χ*^2^ test or Fisher's exact probability method. DFS and OS were calculated using the Kaplan–Meier method and compared by the Log-rank test. *P* < 0.05 indicated statistical significance. IBM SPSS Statistics (version 26.0, SPSS Inc, Chicago, IL) was used for all statistical analyses.

## Results

### Participant and baseline data between the RARS group and NOSES group

All procedures were performed successfully. In the present study, PSM was performed for gender, age, BMI, ASA score, maximum tumor diameter, the height of tumor from the anal verge, histological differentiation, AJCC stage, T stage, N stage, and previous abdominal surgery, and 91 pairs of patients were successfully matched. Afterward, confounding bias was eliminated, and all 91 pairs of participants had comparable baseline information (*P* > 0.05) (Table 1).

**Table 1:** Comparative baseline data in the NOSES group and RARS group before and after PSM.

Baseline characteristics	Before PSM			After PSM		
	RARS (*n* = 144)	NOSES (*n* = 138)	*P*	RARS (*n* = 91)	NOSES (*n* = 91)	*P*
Age at surgery, median (IQR), years	63 (52–71)	60 (52–67)	0.069	63 (52–71)	62 (55–70)	0.749
Sex, *n* (%)			0.013			0.878
Male	100 (69.4)	76 (55.1)		57 (62.6)	58 (63.7)	
Female	44 (30.6)	62 (44.9)		34 (37.4)	33 (36.3)	
ASA score, *n* (%)			0.069			0.830
I/II	116 (80.6)	122 (88.4)		79 (86.8)	78 (85.7)	
III	28 (19.4)	16 (11.6)		12 (13.2)	13 (14.3)	
BMI, mean (SD), kg/m^2^	22.7 (2.8)	22.4 (2.5)	0.337	22.5 (2.8)	22.5 (2.5)	0.954
Maximum tumor diameter, mean (SD), cm	4.4 (1.7)	3.5 (1.5)	0.000	4.0 (1.5)	4.0 (1.5)	0.992
Height of tumor from the anal verge, *n* (%), cm			0.894			0.877
≤15	95 (66.0)	90 (65.2)		59 (64.8)	58 (63.7)	
>15	49 (34.0)	48 (34.8)		32 (35.2)	33 (36.3)	
Histological differentiation, *n* (%)			0.541			0.438
Well	49 (34.0)	44 (31.9)		34 (37.4)	31 (34.1)	
Moderate	83 (57.6)	77 (55.8)		51 (56.0)	49 (53.8)	
Poor	12 (8.3)	17 (12.3)		6 (6.6)	11 (12.1)	
AJCC stage, *n* (%)			0.025			0.217
I	29 (20.1)	35 (25.4)		22 (24.2)	24 (26.4)	
II	45 (31.3)	24 (17.4)		27 (29.7)	17 (18.7)	
III	70 (48.6)	79 (57.2)		42 (46.2)	50 (54.9)	
Pathological T stage, *n* (%)			0.589			0.878
1	16 (11.1)	19 (13.8)		14 (15.4)	11 (12.1)	
2	22 (15.3)	22 (15.9)		15 (16.5)	16 (17.6)	
3	73 (50.7)	74 (53.6)		41 (45.0)	45 (49.4)	
4	33 (22.9)	23 (16.7)		21 (23.1)	19 (20.9)	
Pathological N Stage, *n* (%)			0.077			0.375
0	74 (51.3)	59 (42.8)		49 (53.8)	41 (45.1)	
1	45 (31.3)	61 (44.2)		29 (31.9)	38 (41.8)	
2	25 (17.4)	18 (13.0)		13 (14.3)	12 (13.2)	
Previous abdominal surgery	45 (31.3)	21 (15.2)	0.001	17 (18.7)	18 (19.8)	0.851

Values are presented as mean ± SD, median and IQR (interquartile range), or *n* (%). BMI, body mass index; ASA, American society of anesthesiologists; AJCC, American Joint Committee on Cancer; PSM, propensity score matching; RARS, robotic-assisted resection surgery; NOSES, natural orifice specimen extraction surgery.

### Comparison of perioperative indexes between RARS group and NOSES group

**Figure 2:**
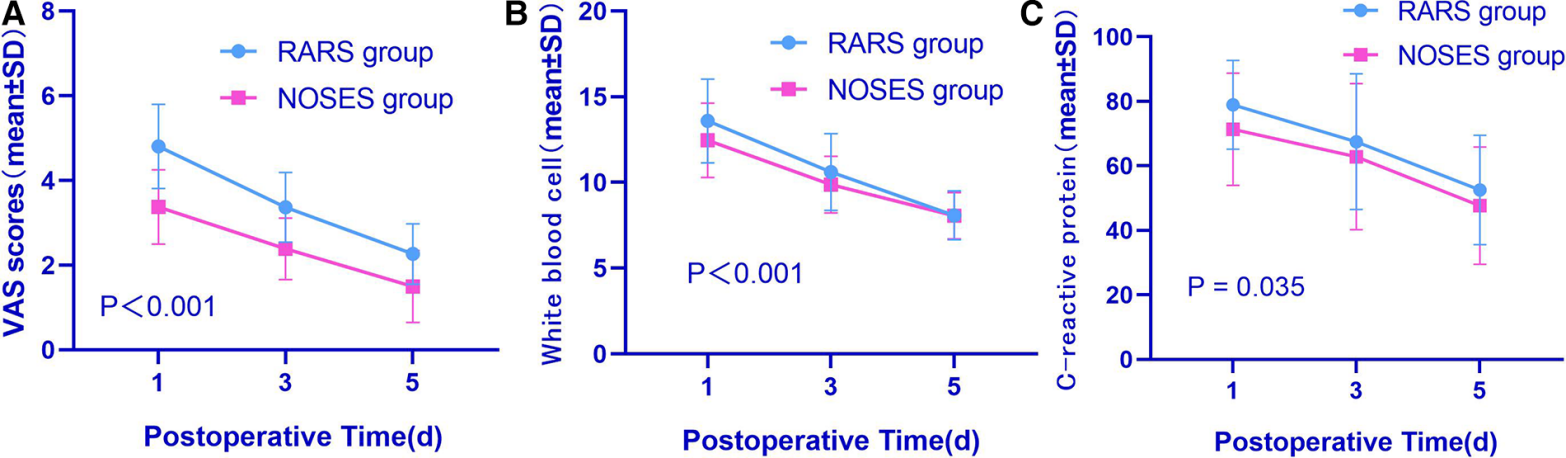
Comparative perioperative indexes in the two groups of patients after PSM (**A–C**). (**A**) VAS scores between two groups; (**B**) white blood cell scores between two groups; (**C**) C-reactive protein scores between two groups. The *P*-value was calculated by repeated measures statistical analysis. VAS, visual analogue scale; RARS, robotic-assisted colorectal cancer resection surgery; NOSES, natural orifice specimen extraction surgery.

As suggested by comparing the perioperative figures, the operative time was essentially the same between both groups (147.1 ± 25.7 min in the RARS group vs. 149.8 ± 31.7 min in the NOSES group, *P* = 0.535). The intraoperative blood loss was similar (54.3 ± 28.0 ml in the RARS group vs. 54.0 ± 30.3 ml in the NOSES group, *P* = 0.949). Gastrointestinal recovery function was better in the NOSES group than that in the RARS group (60.8 ± 16.6 h in the RARS group vs. 54.4 ± 18.1 h in the NOSES group, *P* = 0.014). The abdominal incision length was significantly shorter than that in the RARS group (11.1 ± 0.6 cm in the RARS group vs. 4.8 ± 0.3 cm in the NOSES group, *P* < 0.001). Regarding postoperative recovery indicators, the number of postoperative hospital days was the same in the RARS and NOSES groups (*P* = 0.934) (Table 2). However, compared to the RARS group, the NOSES group reported better pain score results (*P* < 0.001) and had significantly fewer patients requiring additional analgesics (*P* < 0.001). Concerning surgical stress, the white blood cell counts and C-reactive protein levels were compared between the two groups at 1, 3, and 5 days postoperatively. Our results demonstrated that compared to the RARS group, the NOSES group had lower indicators of inflammation (*P* < 0.001, *P* = 0.035) (Figure 2, Table 3). With respect to postoperative complications, nine were observed in the NOSES group, while 13 were in the RARS group. Notably, the NOSES group had fewer wound complications.

**Table 2:** Comparative postoperative conditions in the NOSES group and RARS group.

Outcome	After PSM		
	RARS (*n* = 91)	NOSES (*n* = 91)	*P*
Operative time, mean (SD), min	147.1 (25.7)	149.8 (31.7)	0.535
Estimated blood loss, mean (SD), ml	54.3 (28.0)	54.0 (30.3)	0.949
1st flatus, mean (SD), h	60.8 (16.6)	54.4 (18.1)	0.014
1st oral feeding, mean (SD), h	75.0 (15.3)	70.0 (15.1)	0.026
Postoperative hospital stay, mean (SD), day	9.7 (4.6)	9.6 (4.3)	0.934
Length of abdominal incision, mean (SD), cm	11.1 (0.6)	4.8 (0.3)	<0.001
Postoperative complication, *n* (%)	13 (14.3)	9 (9.9)	0.363
Anastomotic leakage	6 (6.6)	4 (4.4)	
Ileus	1 (1.1)	2 (2.2)	
Wound-related	3 (3.3)	0 (0)	
Urinary retention or infection	0 (0)	1 (1.1)	
Pulmonary infection	1 (1.1)	1 (1.1)	
Others	2 (2.2)	1 (1.1)	
Harvested lymph nodes, *n* (%)			0.188
≤12	21 (23.1)	14 (15.4)	
>12	70 (76.9)	77 (84.6)	
Harvested positive lymph nodes, mean (SD)	1.1 (1.8)	0.98 (1.3)	0.565
Perineural invasion, *n* (%)	36 (39.6)	38 (41.8)	0.763
Lymphatic or vascular invasion, *n* (%)	26 (28.6)	38 (41.8)	0.062
Positive margin	0 (0)	0 (0)	NA
Postoperative PFDI-20 scores, mean (SD)	7.4 (1.3)	7.1 (1.4)	0.152

Values are presented as mean ± SD or *n* (%). PSM, propensity score matching; RARS, robotic-assisted resection surgery; NOSES, natural orifice specimen extraction surgery.

**Table 3:** Comparison of postoperative stress response and pain condition of patients between the NOSES group and RARS group.

Variable	After PSM		
	RARS (*n* = 91)	NOSES (*n* = 91)	*P*
Postoperative white blood cell, mean (SD), count/L			<0.001*
Day 1	13.6 (2.4)	12.5 (2.2)	
Day 3	10.6 (2.2)	9.8 (1.7)	
Day 5	8.1 (1.4)	8.1 (1.4)	
Postoperative C-reactive protein, mean (SD), mg/L			0.035*
Day 1	79.0 (13.8)	71.3 (17.4)	
Day 3	67.5 (21.1)	62.9 (22.7)	
Day 5	52.5 (16.9)	47.7 (18.2)	
VAS scores, mean (SD)			<0.001*
Day 1	4.8 (1.0)	3.4 (0.9)	
Day 3	3.4 (0.9)	2.4 (0.7)	
Day 5	2.3 (0.7)	1.5 (0.8)	
Usage of additional analgesics, *n* (%)	37 (40.7)	13 (14.3)	<0.001

Values are presented as mean ± SD. VAS, visual analogue scale; PSM, propensity score matching; RARS, robotic-assisted resection surgery; NOSES, natural orifice specimen extraction surgery.

* The *P*-value was calculated by repeated measures statistical analysis.

### Comparison of short-term quality of life between RARS group and NOSES group

**Figure 3:**
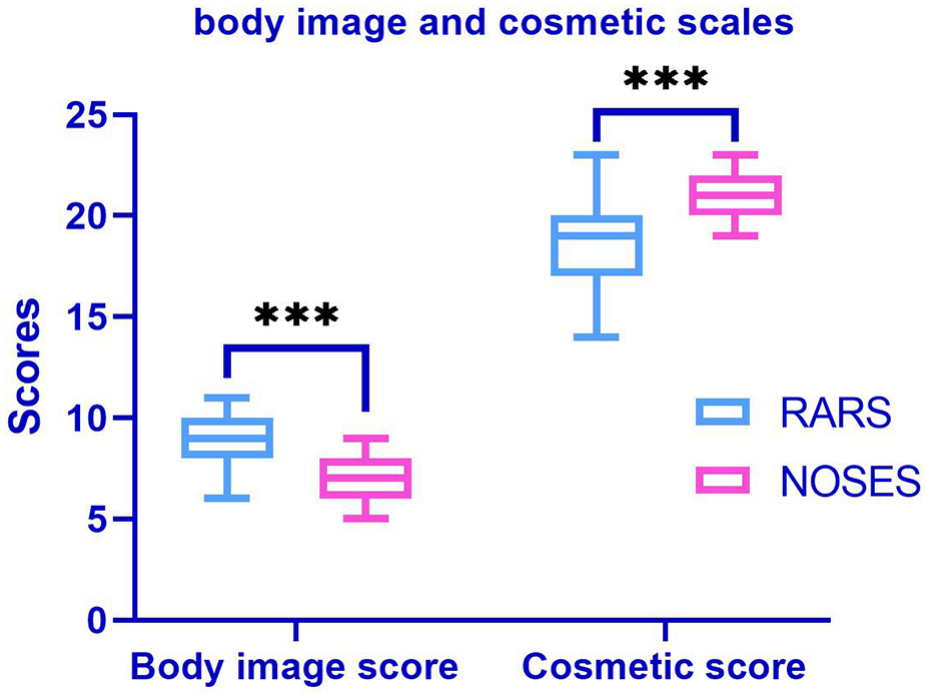
Comparative body imagery questionnaire (BIQ) in the two groups of patients after PSM. The body image score (among 5 and 20, a lower score means better body image). Cosmetic score (among 3 and 24, a higher score means better cosmetic results). ***P < 0.001. RARS, robotic-assisted colorectal cancer resection surgery; NOSES, natural orifice specimen extraction surgery.

**Figure 4:**
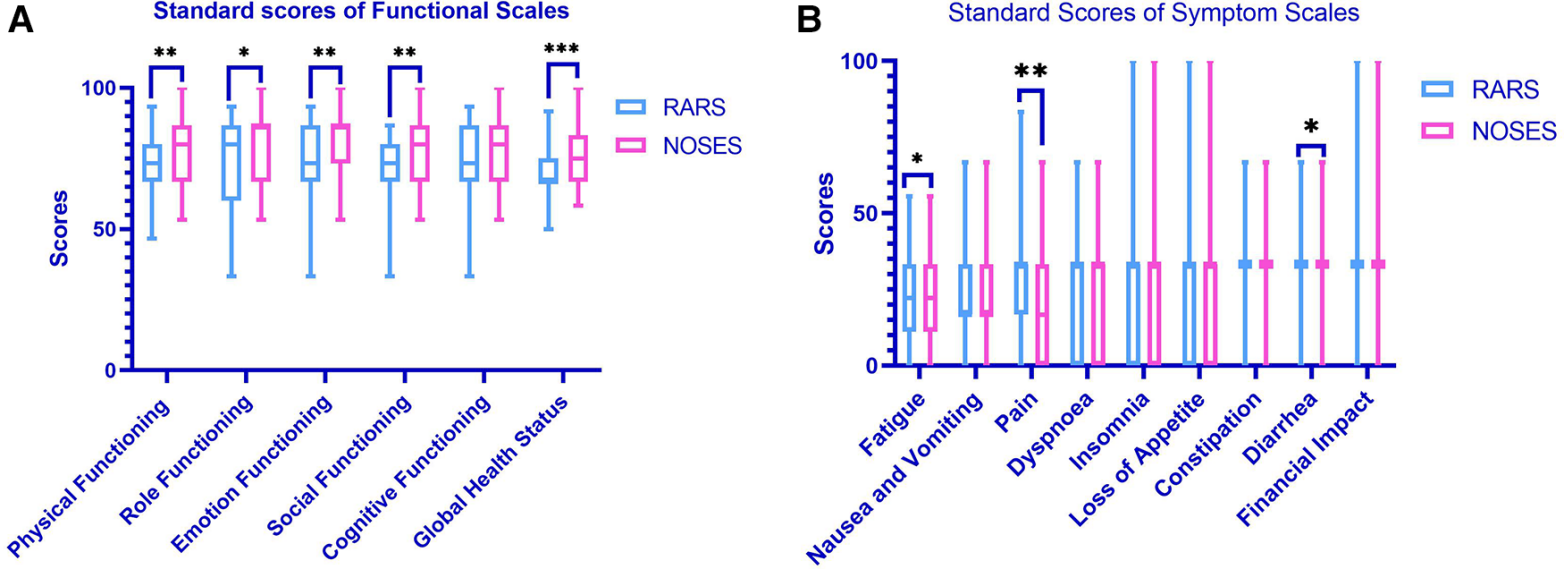
Comparative EORCT quality of life questionnaire-core 30 results in the two groups of patients after PSM (**A,B**). (**A**) Functional scales between two groups (a higher score means better functional results); (**B**) symptom scales between two groups (a lower score means better symptom results). *P < 0.05, **P < 0.01, ***P < 0.001. RARS, robotic-assisted colorectal cancer resection surgery; NOSES, natural orifice specimen extraction surgery.

At 1 month postoperatively, the NOSES group had significantly better body imagery scores (*P* < 0.001) and cosmetic outcomes (*P* < 0.001) than the RARS group (Figure 3). The PFDI-20 scores of the NOSES and RARS groups were not significantly different 3 months after surgery. Besides, somatic function (*P* = 0.003), role function (*P* = 0.039), emotional function (*P* = 0.001), social function (*P* = 0.004), and overall function (*P* < 0.001) in the NOSES group were significantly better than those in the RARS group (Figure 4). The NOSES group had less fatigue (*P* = 0.024), pain (*P* = 0.004), and diarrhea (*P* = 0.044) at 3 months postoperatively. Additionally, the PASQ Total Subscale Score and Global Subscale Score at 3 months postoperatively in the NOSES group were significantly lower than those in The RARS group (Table 4). Nonetheless, the RARS and NOSES groups maintained an identical standard of anal function at the 6-month postoperative evaluation of anal functional capacity (Table 5).

**Table 4:** Comparative PSAQ responses of patients between the NOSES group and RARS group.

Subscale	Total subscale score			Global subscale score		
	RARS (*n* = 91)	NOSES (*n* = 91)	*P*	RARS (*n* = 91)	NOSES (*n* = 91)	*P*
Appearance	14 (13–17)	11 (10–14)	<0.001	3 (1–3)	1 (1–2)	0.001
Symptoms	10 (8–12)	7 (7–9)	<0.001	2 (2–3)	1 (1–2)	<0.001
Scar consciousness	8 (7–10)	7 (6–9)	0.014	2 (1–3)	2 (1–3)	0.001
Satisfaction with appearance	10 (9–13)	9 (8–9)	<0.001	2 (2–3)	2 (1–2)	<0.001
Satisfaction with symptoms	7 (6–9)	8 (7–9)	0.451	2 (2–3)	2 (1–3)	<0.001
Total	50 (46–61)	44 (40–48)	<0.001	12 (10–13)	9 (7–10)	<0.001

Values are presented as median and IQR (interquartile range). PSAQ, patient scar assessment questionnaire and scoring system; RARS, robotic-assisted resection surgery; NOSES, natural orifice specimen extraction surgery.

**Table 5:** Comparative postoperative wexner scores of patients between NOSES group and RARS group.

Type of incontinence	After PSM		
	RARS (*n* = 91)	NOSES (*n* = 91)	*P*
Solid	1 (1–3)	1 (1–2)	
Liquid	2 (1–2)	2 (1–3)	
Gas	2 (1–3)	3 (1–3)	
Wears pad	0 (0–1)	1 (0–2)	
Lifestyle alteration	2 (1–2)	1 (1–2)	
Total score	9 (4–10)	9 (5–10)	0.086

Values are presented as median and IQR (interquartile range). PSM, propensity score matching; RARS, robotic-assisted resection surgery; NOSES, natural orifice specimen extraction surgery.

### Long-term survival outcomes between RARS group and NOSES group

**Figure 5:**
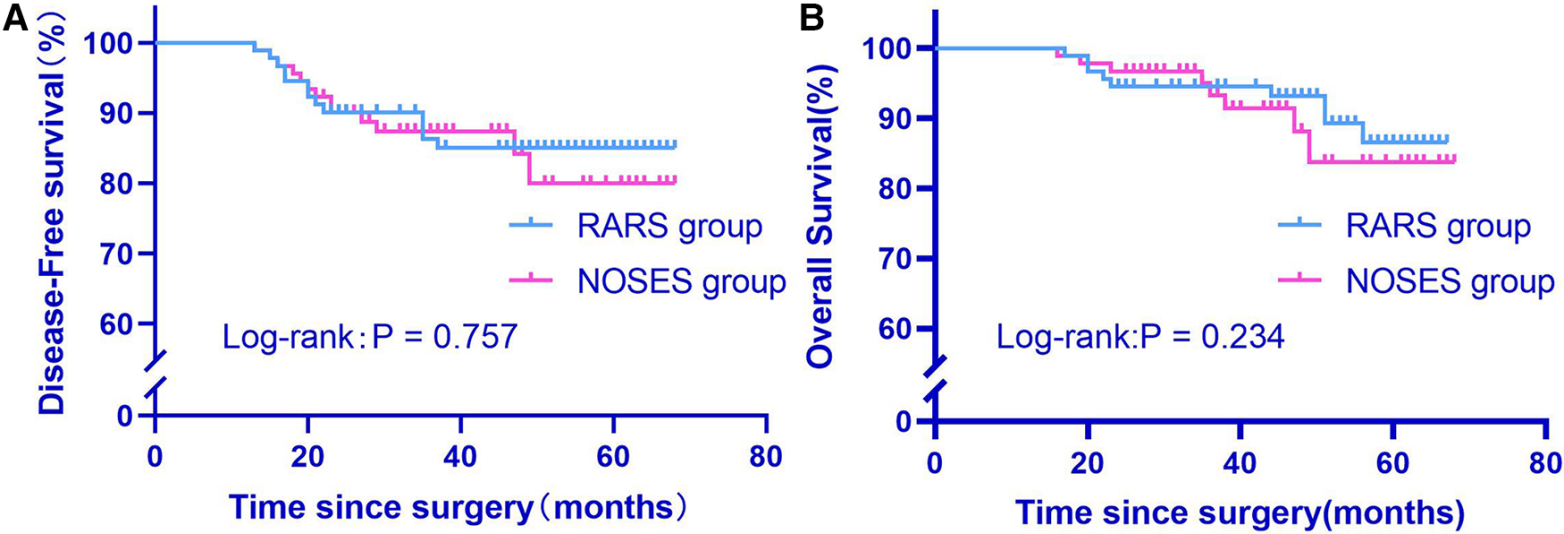
Comparison of overall survival and disease-free survival between two groups after propensity score matching (**A,B**). RARS, robotic-assisted colorectal cancer resection surgery; NOSES, natural orifice specimen extraction surgery.

At the last follow-up as of September 31, 2022, the median follow-up was 51 months (17–67) and 38 months (16–68) for the RARS group and the NOSES group, respectively. The 2-year overall survival rate was 94.5% and 96.7% in the RARS group and the NOSES group, respectively. The Log-rank test revealed no statistically insignificant difference in overall survival between the two groups (*P* = 0.234). Furthermore, both groups were not significantly different in 2-year disease-free survival (*P* = 0.757) (Figure 5).

## Discussion

Given the continuous advancement of minimally invasive technology, the surgery for radical colorectal cancer has changed from traditional open-to-laparoscopic surgery to present-day robotic surgery with the coexistence of all three (21). In 2000, the FDA approved the use of robotic surgery systems in clinical practice, leading to yearly increasing reports of robotic NOSES for colorectal cancer (22–24). Currently, most of the studies are retrospective, and there are confounding factors influencing the authenticity of some of the findings (25). This study applied propensity score matching to effectively weaken the group's confounding effect and balance the differences. The results were more objective, realistic, and comparable.

Surgical safety is the primary prerequisite for performing robotic colorectal cancer NOSES. Efetov et al.'s (26) findings concluded that NOSES has the benefit of reduced intra-abdominal infection potential risk. Our study strictly adhered to the principles of aseptic surgery during the operation, removed the specimen using a sterile-protected package, disinfected it promptly, and flushed the abdominal cavity with plenty of salines. Regarding postoperative complications, the intra-abdominal infections in the NOSES and the RARS groups did not differ significantly. Meanwhile, the mean operative time and bleeding volume were similar in the two groups, consistent with Liu et al. (27). Concerning the tumor-free principle, no difference between groups was observed in the number of lymph nodes cleared and the percentage of positive tumor margins. This was attributed to the broader field of view and flexible robotic arm of the robotic surgical system. These findings further confirmed that robotic NOSES for colorectal cancer has surgical efficacy that is not inferior to robotic-assisted colorectal cancer resection.

The short-term outcome of surgery is a crucial indicator to evaluate the quality of robotic colorectal cancer NOSES. Since the length of the abdominal wall incision is noticeably shorter with robotic NOSES, patients also experience considerably less postoperative pain. They require less additional analgesia, and patients can get up and move around early. Therefore, the restoration of gastrointestinal function was earlier for the NOSES group than for the RARS group. Similarly, the leukocyte indicators and C-reactive protein levels of the two groups were compared at 1, 3, and 5 days postoperatively. Particularly, surgical stress may promote the growth of preexisting micrometastases or trigger tumor growth (28, 29). Inflammatory indexes in the NOSES group were lower than those in the RASR group, suggesting that the NOSES group had less organismal disturbance and a more pronounced minimally invasive advantage for the patients.

Most current retrospective studies often overlooked the postoperative quality of life assessment. The cosmetic results of NOSES are also one of the reasons for patients choosing this procedure, especially young female patients. When surgical incision complications or scarring were expected to heal fully, the patients’ body imagery and cosmetic outcomes were explored, starting with their feelings. It was demonstrated that body imagery and cosmetic outcomes in the NOSES group were significantly better than those in the RARS group. The findings were consistent with the PSAQ scale. The EORTC QLQ—C30 scale, divided into a functional scale and a symptom scale, has been used in breast cancer (30) and prostate cancer (31). The EORTC QLQ—C30 scale was divided into functional and symptom scales, validated in breast and prostate cancer. The results implied that the NOSES group had better results than the RARS group. This could be explained that the more prominent the postoperative surgical scar, the more negative the patient's psychological cues about cancer treatment, and the greater the irritability and panic, the worse the results provided by the EORTC QLQ—C30 scale. Some researchers believe that injury to anal function may occur during transanal removal of the specimen and anastomosis of the intestinal canal (32, 33). In the indications for NOSES (34), the patient's BMI and tumor size were strictly controlled. The anal sphincter function was not damaged during the procedure. Thus, the anal function was kept at the same level in both groups regarding the Wexner score. Moreover, no significantly different OS and DFS for the two groups were observed concerning long-term oncologic outcomes.

This study also has some limitations. First, there will be a certain amount of selection bias as this is a retrospectively studying. Hence, propensity score matching was selected to reduce variance. Secondly, the sample size was insufficient due to the slight size limitation of a single-center study. It is ensured in this study that the same surgeon-led specialty team performs all procedures, so as to curtail variability from background or surgical skills among surgeons. For this reason, our center is conducting a multicenter, prospective randomized controlled study of robotic NOSES (35) to lay a solid foundation of robotic NOSES for colorectal cancer to guide the surgical treatment of colorectal cancer better.

## Conclusion

Robotic colorectal cancer NOSES is a safe and feasible minimally invasive technique and offers shorter abdominal incisions, less pain, less surgical stress response, and better postoperative quality of life. Therefore, this technique can be further promoted for colorectal cancer patients eligible for NOSES.
